# Supplementary material for: Geometric Morphometric Wing Analysis of Avian Malaria Vector, Culiseta longiareolata, from Two Locations in Algeria
Source: Insects. 2022 Nov 8;13(11):1031. doi: 10.3390/insects13111031 (PMC9693553; doi:10.3390/insects13111031)
Supplement: Supplementary file 1 [file insects-13-01031-s001.zip › insects-1943296-supplementary.pdf]

Supplemental Table S1. Information of *Cs. Longiareolata* mosquito samples from each study area. The weather data from the actual sampling year, altitude, geographic coordinates, type of climate, type of habitat, and the number of mosquito individuals per sex.

District		T (°C)	R (mm)	A (m)	Geographic coordinates		Climate	Larval habitat type	Number of mosquito samples	
					**North**	**East**			**Male**	**Female**
**GUELMA (AinMakhlouf)**	AM01	15,3	502,1	791	36°15'30.93"	7°16'38.61"	Sub-arid	Natural	08	35
	AM02	15,3	502,1	818	36°12'42.03"	7°12'57.75"				
	AM03	15,3	502,1	846	36°14'12.37"	7°14'49.38"				
	AM07	15,3	502,1	863	36°16'54.05"	7°16'50.22"				
	AM04	15,3	502,1	825	36°14'22.61"	7°15'03.43"		Artificial	20	28
	AM05	15,3	502,1	825	36° 14'50.10"	7°15'08.56"				
**ANNABA & EL TARF**	CH 01	17,2	947,3	65	36° 41'43.43"	7°34'12.84"	Sub-humid	Natural	18	38
	AS01	17,2	947,3	55	36° 41'25.24"	7°39'07.83"				
	SK	17,8	1223	14	36° 45'24.87"	7°58'19.22"				
	BM	17,8	1223	3	36°46'19.11"	7°53'50.82"				
	SA	17,2	947,3	15	36° 48'53.41"	7°43'26.72"		Artificial	42	76
	AS02	17,2	947,3	55	36° 41'25.24"	7°39'07.83"				
	SEB02	17,8	1223	5	36° 50'22.97"	8°03'49.15"				

T: temperature; **R**: rainfall; **A**: altitude; **AM**: Ain Makhlouf; **CH**: Chorfa; **AS**: Ain sayed; **SA**: Sidi Amar; **SK**: Sidi kassi; **BM**: Ben Mhidi; **SEB**: Sebaa

Supplementary Table S2. Corresponding description and location of the wing and landmark positions in

Cs. Longiareolata.

**Landmarks**	**Description of the landmarks**
**1**	Radial sector
**2**	Intersection of costa
**3**	Distal end of radius
**4**	Radial branch 2
**5**	Radial branch 3
**6**	Distal end of radial branches 4 and 5
**7**	Distal end of media 1 and 2
**8**	Distal end of media 3 and 4
**9**	Distal end of cubital vein 1
**10**	Distal end of cubital vein 2
**11**	Anal vein
**12**	Origin of cubital 1
**13**	Midpoint branch of cubital 3
**14**	Medio-cubital cross vein
**15**	Midpoint branch of medial vein
**16**	Radio-sectoral vein
**17**	Radio-medial cross vein
**18**	Midpoint branch of radial vein
**19**	Origin of radius branches 2 and 3

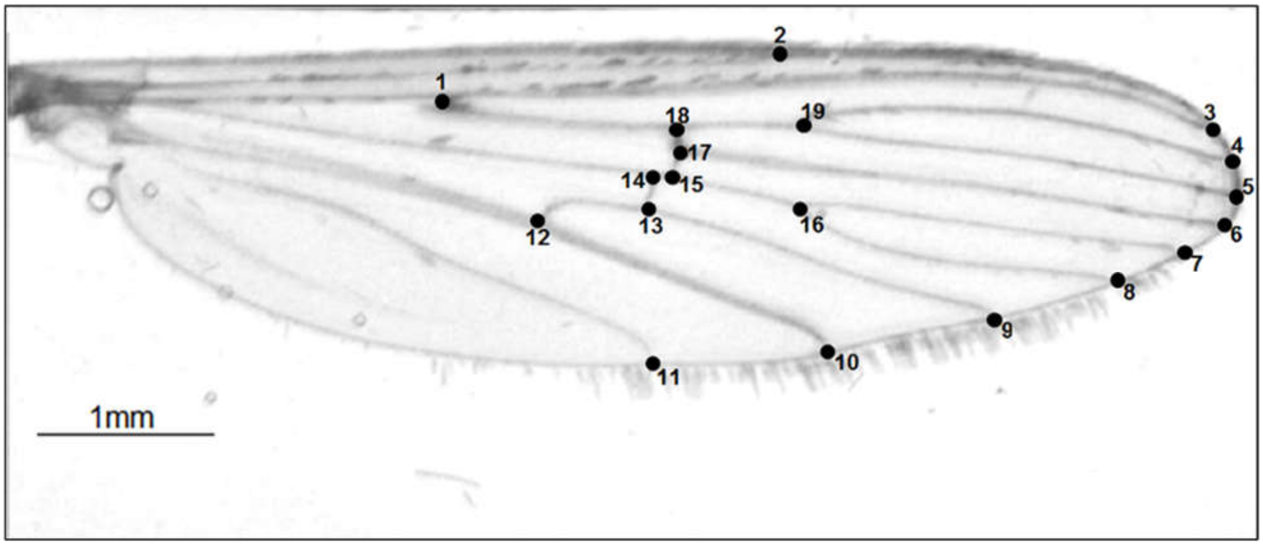

Supplemental Figure S1. Digitization of 19 landmarks of the right wing of a female *Culiseta longiareolata* using software Tps-Dig2 2.31.
